# Ausgabenspielräume der Bundesregierung: zwischen Schuldenbremse und Steuererhöhung

**DOI:** 10.1007/s10273-021-3002-6

**Published:** 2021-09-21

**Authors:** Matthias Diermeier, Michael Hüther, Thomas Obst

**Affiliations:** Institut der deutschen Wirtschaft Köln e.V., Konrad-Adenauer-Ufer 21, 50668 Köln, Deutschland

**Keywords:** H3, H6, H61, H68

## Abstract

Die neue Bundesregierung wird zur Finanzierung neuer Ausgabenschwerpunkte nur bedingt auf eine Kreditfinanzierung ausweichen können, da nach aktuellem Stand ab 2023 wieder die Schuldenbremse greifen wird. Auch die Möglichkeit, die Mehrausgaben durch Steuererhöhungen zu decken, kann als schwieriges Unterfangen gewertet werden. Die Autoren haben mit Blick auf die Wahlprogramme der Parteien die budgetären Spielräume im Bundeshaushalt für die kommende Legislaturperiode genauer betrachtet. Sie schlussfolgern, dass ohne weitere Schuldenfinanzierung oder Steuererhöhungen der Bundeshaushalt bei heutiger Planung an seine verfassungsrechtlichen Kreditaufnahmegrenzen stoßen wird.

## Abstract (en)

The 2021 election campaign in Germany has revealed substantial demands on the federal budget in the coming legislative period. Some coalition partners, however, have rejected a reform of the debt brake and simultaneously promise tax cuts. A ‘burden forecast’ for the next four years indicates that these campaign promises will triple expenditures from 37 to 105.1 billion euro and will exclude urgent investments to modernise public infrastructure, cope with climate change and tackle digital transformation. With the debt brake maxed out, the financing of up to 263 billion euro remains up in the air. Keeping the debt brake without increasing taxes would be like trying to ‘square the circle’.

In Wahlkampfzeiten gießen Parteien ihre Zukunftsvisionen in Programme; daran hängen Preisschilder – manchmal direkt ausgezeichnet, wenn Entlastungen oder Ausgabenversprechen konkretisiert werden sollen, oft jedoch nur indirekt ermittelbar, da unvermeidbare Inkonsistenzen infolge unzureichender Orchestrierung gruppenspezifischer Forderungen überdeckt werden sollen. Auch beim Warmlaufen für die Bundestagswahl, die nicht zuletzt im Zeichen der über 500 Mrd. Euro pandemiebedingten Nettokreditaufnahme in den Jahren 2020, 2021 und 2022 steht, wird man in den vorgelegten Programmen fündig.

Die Grünen fordern ein „Investitionsprogramm“ (Bündnis 90 / Die Grünen, 2021, 27) für den Klimaschutz sowie eine Teilerstattung der teuren EEG-Umlage über ein „Energiegeld“ (Bündnis 90 / Die Grünen, 2021, 20). Die FDP zielt auf die Nachholbedarfe in der Digitalisierung ab, stellt die „digitale Modernisierung“ (FDP, 2021a, 23) des Staates in den Vordergrund und verspricht umfangreiche Steuersenkungen. Die CDU/CSU misst der transatlantischen Partnerschaft einen besonderen Wert bei und bekennt sich zum 2 % Ausgabenziel der Verteidigungsgemeinschaft (CDU/CSU, 2021, 9). Zudem sieht die Union vor, die Sozialleistungsquote für Arbeitgeber:innen und Arbeitnehmer:innen bei 40 % zu deckeln (CDU/CSU, 2021, 34), eine Positionierung, der auch SPD und FDP folgen. Die CSU klammert sich an die „Mütterrente“ (CSU, 2021, 2) und macht diese gleich zur – einzigen – Voraussetzung für eine mögliche Regierungskoalition (Tagesschau, 2021). Die SPD erklärt das Tätigen öffentlicher Investitionen zu ihrer „Mission“ (SPD, 2021, 8) und möchte gleichzeitig den Sozialstaat ausbauen. Die EEG-Umlage in den Bundeshaushalt zu überführen, trifft weitgehend bei diesen Parteien auf Zustimmung.

Zur Finanzierung neuer Haushaltsschwerpunkte wird die neue Bundesregierung aufgrund der – nach aktuellem Stand ab 2023 – wieder greifenden Schuldenbremse nur sehr bedingt auf eine Kreditfinanzierung ausweichen können. Das bedeutet im Umkehrschluss: Mehrausgaben respektive Einnahmenausfälle müssten durch Steuereinnahmen gedeckt werden. Bei den großen Zielen, die sich die unterschiedlichen Parteien gesetzt haben, darf dies durchaus als schwieriges Unterfangen eingeordnet werden, zumal zwei Parteien – Union und FDP – Steuersenkungen für alle in Aussicht stellen. Da sich bei allen zitierten Parteien die Überzeugung durchgesetzt hat, dass die lange überfällige Entlastung von mittleren Einkommen im Vordergrund stehen soll, werden steuerlich kaum nennenswerte Aufkommenseffekte zu holen sein. Während der SPD-Vorschlag über eine stärkere Besteuerung höherer Einkommen noch minimal positive Nettoaufkom-

**Matthias Diermeier**, MA in Economics, und **Dr. Thomas Obst**, sind persönliche Referenten des Direktors am IW in Köln.

**Prof. Dr. Michael Hüther** ist Direktor des IW in Köln und Honorarprofessor an der European Business School.

menseffekte veranschlagt, ist die Grünen-Steuerreform aufkommensneutral konzipiert. Mindereinnahmen hätte der künftige Bundeshaushalt zu verkraften, würden CDU/CSU oder FDP nach der Wahl ihre Überlegungen umsetzen: Dann lägen die aggregierten Mindereinnahmen bei 20 Mrd. Euro beziehungsweise 75 Mrd. Euro jährlich (Beznoska und Hentze, 2021).

So offenbaren die in den Wahlprogrammen der unterschiedlichen Parteien vorgelegten Ausgabenvorschläge und Einnahmepläne ihre Inkonsistenzen. Höhere Ausgaben, bei konstanten oder sinkenden Steuereinnahmen lassen sich ohne eine Reform der Schuldenbremse kaum umsetzen. Unangenehm deutlich ist dieser Befund bereits Kanzlerkandidat Armin Laschet aufgestoßen, der in derselben Woche, in der das CDU/CSU Wahlprogramm veröffentlicht wurde, konstatieren musste: „Für Steuererleichterung haben wir nicht das Geld“ (Handelsblatt, 2021).

Der folgende Beitrag beleuchtet, wie deutlich Soll und Haben im Bundeshaushalt der kommenden Jahre auseinanderklaffen könnten. Damit wird deutlich, worum es bei den fiskalisch wirksamen Vorschlägen der Wahlprogramme geht: um dauerhafte, mithin strukturelle Veränderung und nicht um einmalige Belastungen oder konjunkturabhängige Wünsche. Deshalb wird im Weiteren von der konjunkturellen Schwankung im Bundeshaushalt abstrahiert, deren Effekte auf die Kreditaufnahme im Rahmen der Schuldenbremse ohnehin grundsätzlich unproblematisch sind. Ebenso werden Einmaleffekte – als Finanzierungspotenziale wie die „Rücklage Asylbewerber und Flüchtlinge“ (48 Mrd. Euro) oder als Belastung wie der Wiederaufbaufonds für die Flutschäden (für den Bund mit 16 Mrd. Euro beschlossen) – in den Sondervermögen des Bundes (Bundesfinanzministerium, 2019) nicht berücksichtigt.

## Sturm im Paradies: Normalität nach dem Ausnahmezustand?

Das Loch, das die Ausgaben zur Bekämpfung der Pandemiefolgen in den Bundeshaushalt gerissen haben, ist beachtlich (vgl. Abbildung 1). Seit 2012 bis zum Pandemiebeginn hatte der deutsche Staat keine nennenswerten Finanzierungsdefizite mehr über eine Kreditaufnahme am Kapitalmarkt ausgleichen müssen (in der Grafik ab 2015 dargestellt). Mit wachsendem Bruttoinlandsprodukt (BIP)reduzierte sich die Schuldenstandquote gemäß Maastrichtkriterium von ihrem Höchststand knapp über 80 % im Jahr 2010 auf schließlich 59,6 % des deutschen BIP im Jahr 2019 (Bundesfinanzministerium, 2021a). Inwiefern die ab 2016 auf Bundesebene greifende Schuldenbremse ihren Beitrag zur deutschen Staatsschuldenkonsolidierung geleistet hat und wie sinnvoll ihre Beibehaltung angesichts der vielschichtigen Dimensionen des

Abbildung 1
**Entwicklung Ausgaben und Einnahmen des Bundes**
in Mrd. Euro

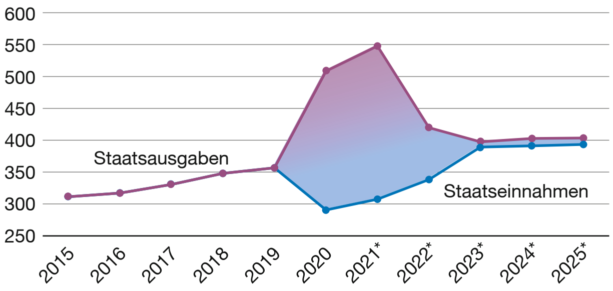

* Werte beziehen sich auf den Finanzplan des Bundes von 2020 bis 2024.

Quelle: Bundesfinanzministerium (2021b).

Strukturwandels ist, denen sich die deutsche Wirtschaft gegenübersieht, darüber wird intensiv diskutiert (Hüther, 2019; Hüther und Südekum, 2020; Feld et al., 2020).

In jedem Fall haben sich die Rahmenbedingungen der Staatsfinanzen aufgrund der enormen Kosten und Einnahmenausfälle, die aus der Bekämpfung der COVID-19-Pandemie resultierten, seit 2019 grundlegend verändert. In den nur drei Jahren, während derer die Schuldenbremse nach heutigem Stand ausgesetzt sein wird, plant der Bund eine Nettokreditaufnahme von zusammengenommen 540 Mrd. Euro. 2023 sollen sich die Ausgaben schließlich schuldenbremsenkonform wieder den geschätzten Einnahmen annähern. Ab 2023 sieht die Eckwerteplanung des Finanzplans 2021 bis 2025 allerdings bereits heute eine weitere Verschuldung in Höhe von rund 10 Mrd. Euro jährlich vor (Bundesfinanzministerium, 2021b). Selbst wenn die strukturelle Komponente der Schuldenbremse maximal ausgereizt werden sollte, ließe der Neuverschuldungsspielraum – bei 0,35 % des BIP umgerechnet rund 12 Mrd. bis 14 Mrd. Euro – allerdings kaum die im Bundeswahlkampf diskutierten weiteren Ausgaben zu.

Die vielfältigen, strukturell wirksamen Wahlversprechen der unterschiedlichen Parteien im Vorfeld der Bundestagswahl stehen angesichts der bereits ausgereizten Ausgabenspielräume im Bundesfinanzplan vor einem verfassungsrechtlichen Finanzierungsvorbehalt. Zwar unterliegen die Vorschläge und ihre Bepreisung (noch) großer Unsicherheit, eine entsprechend annahmenbehaftete Kostenkalkulation zeigt jedoch eindrücklich, wie deutlich das strukturelle Neuverschuldungsmaximum im Sinne der Schuldenbremse verfehlt werden würde (vgl. Tabelle 1). Wer die grundgesetzliche Schuldenbremse unangetastet lassen will und damit gar eine wahlprogrammatische Position verbindet, der muss sich die Frage stellen

Tabelle 1
**Mögliche zusätzliche Belastungen des Bundeshaushalts**

| In Mrd. Euro | 2022 | 2023 | 2024 | 2025 |
|---|---|---|---|---|
| Zusätzliche Ausgaben | 36,96 | 62,39 | 82,94 | 105,06 |
| Mütterrente[1] | 4,1 | 4,1 | 4,1 | 4,1 |
| EEG[2] | 16,07 | 19,73 | 18,38 | 18,38 |
| Verteidigung[3] | 8,46 | 14,94 | 21,77 | 28,97 |
| Sozialbeitragsdeckel 40 % | 1,54 | 16,02 | 30,79 | 45,82 |
| Abschaffung Soli[4] | 6,8 | 7,6 | 7,9 | 7,8 |
| Ausgaben gesamt | 456,76 | 459,89 | 485,64 | 508,46 |

Anmerkungen: [1] Mütterrente mit Anrechnung von drei Rentenpunkten für Eltern von vor 1992 geborenen Kindern (Forderung CSU). [2] Förderkosten abzüglich bereits einkalkulierter EEG-Strompreisentlastung im Finanzplan 2020. Wert im Jahr 2025 entspricht Vorjahreswert. [3] Linearer Anstieg von 1,4 % (2020) auf 2 % vom BIP bis 2024 abzüglich der bereits geplanten Verteidigungsausgaben. Bereits eingeplante Ausgaben wurden von Bardt (2021) übernommen. [4] Sozialbeiträge und Beitragsquote 2021 und 2022 IW-Konjunkturprognose, ab 2022 linear fortgeschrieben nach BDA-Kommission (2020).

Quelle: eigene Zusammenstellung und Berechnung.

lassen, wie er denn die gleichermaßen vorgetragenen Ausgabenvorschläge damit in Einklang bringen will.

Die Einführung der dritten Stufe der Mütterrente, die der Bayerische Ministerpräsident Markus Söder zur Bedingung für eine Regierungskoalition gemacht hat, würde nach Aussage der Rentenversicherung z. B. Kosten in Höhe von rund 4,1 Mrd. Euro jährlich verursachen (RND, 2021). Dies wäre dann der Fall, wenn die „Mütterrente II" um eine Anrechnung von 3 anstatt 2,5 Rentenpunkten für Eltern von vor 1992 geborenen Kindern erweitert werden würde. Da der demografische Wandel die Rentenkassen schon ohne Mütterrente an die Belastungsgrenze treibt, scheint ein Überwälzen der Finanzierungskosten auf den Bundeshaushalt unumgänglich, möchte man die Finanzierungsschwierigkeiten der Rentenversicherung nicht noch weiter verschärfen. Ein systematisches oder gerechtigkeitsbezogenes Argument für die Weiterführung der Mütterrente gibt es ohnehin nicht; der Vorschlag reflektiert allein die erwartete Wählermobilisierung.

Noch wesentlich kostspieliger stellt sich eine Reform der EEG-Umlage dar, die allerdings systematisch – energie- und klimapolitisch – gut zu begründen ist. Die Einspeisegarantien für Betreibende von (hauptsächlich älteren) erneuerbaren Energiequellen macht aktuell etwa ein Fünftel des Haushaltsstrompreises aus und belastet gerade Haushalte mit geringeren Einkommen empfindlich. Eine Übernahme der Umlage durch den Bundeshaushalt ließe sich demnach auch aus Verteilungsgesichtspunkten rechtfertigen. Die erwarteten akkumulierten Förderkosten betragen 2022 rund 20 Mrd. Euro, ab 2023 (wenn sich die Umlage wie erwartet auf 8 Cent pro kWh einpendelt) rund 25 Mrd. Euro. Diese Prognose unterliegt der Annahme, dass die Bundesregierung ihre aktuellen Förderziele entsprechend der EEG-Novelle 2021 erfüllt. Abzüglich der bereits im Finanzplan verankerten Strompreisentlastungen (Bundesregierung, 2020) ergeben sich noch nicht im Finanzplan hinterlegte Belastungen des Bundehaushalts in Höhe von 16 Mrd. bis 18 Mrd. Euro jährlich.

Eine empfindliche zusätzliche Belastung kommt auf die zukünftige Bundesregierung zudem zu, sollte Deutschland seiner Zusage an die NATO-Partner gerecht werden und die Verteidigungsausgaben von 1,4 % 2020 sukzessive bis auf 2 % des BIP im Jahre 2025 anheben. Nimmt man einen linearen Anstieg des bislang mit konstantem Volumen geplanten Rüstungsetats an (Bardt, 2021), würden die zusätzlichen Ausgaben (gegeben den im Finanzplan angesetzten Projektionen des BIP-Wachstums) von 8,5 Mrd. Euro auf 29 Mrd. Euro im Jahr 2025 ansteigen. Dieser Punkt ist zwar zwischen den Parteien umstritten, er ist aber mit Blick auf die internationale Position der Bundesrepublik und die grundsätzlich von fast allen Parteien akzeptierte sicherheitspolitische Verantwortung sowie die transatlantische Partnerschaft letztlich unvermeidbar.

Demografiebedingt werden zudem die Belastungen der Sozialkassen sprunghaft ansteigen. Modellierungen der BDA-Kommission Zukunft der Sozialversicherungen (2020) zeigen bereits für 2025 einen Anstieg der Sozialbeiträge in Prozent der beitragspflichtigen Einkommen auf 42,9 %. Dem entspräche ein Volumen von grob gerechnet bis zu 45,8 Mrd. Euro, dass die Arbeitnehmer:innen und Arbeitgeber:innen zusätzlich aufzubringen hätten. Nimmt man die Forderung der CDU/CSU ernst, die Beiträge bei 40 % deckeln zu wollen, steht der Bundeshaushalt vor nochmals deutlich ansteigenden Belastungen. Denn eine teilweise diskutierte Quersubventionierung aus weniger belasteten an mehr belastete Sozialkassen vermag den demografisch bedingten Anpassungsdruck nicht aufzulösen, geschweige denn den notwendigen Strukturwandel dauerhaft zu entlasten. Um das Beitragssatzziel dennoch zu erreichen, wären deshalb entschieden höhere Steuerzuschüsse vonnöten. Der naheliegende Payer of Last Resort wäre also das Bundesfinanzministerium, das – einen linearen Anstieg der Sozialbeiträge angenommen – die 2025 auf 45,8 Mrd. Euro anwachsende Lücke ausgleichen müsste. Dabei läge in der Verlängerung der beitragspflichtigen Lebenserwerbszeit ein großer Reformhebel, die Sozialversicherungen auch langfristig zu entlasten.

Dies stößt aber nur bei der FDP auf Zuspruch, bei allen anderen Parteien indes auf strikte Ablehnung.

Auf der Einnahmenseite könnte den Bundeshaushalt zudem das von der FDP ins Auge gefasste Abräumen des Rest-Solidaritätsbeitrags belasten. 2019 hatte die Bundesregierung diesen bereits für breite Teile der Bevölkerung abgeschafft. Seitdem wird der „Soli" jedoch weiterhin für rund 6 Mio. Personen und 500.000 Kapitalgesellschaften fällig; er ist quasi zu einer Unternehmenssondersteuer geworden. Simulationsrechnungen deuten auf Nettomindereinnahmen von jährlich 7 Mrd. bis 8 Mrd. Euro hin, die dem Bundeshaushalt nicht mehr zur Verfügung stünden (Hentze und Kolev, 2020). Hierbei werden wachstums- und steuereinnahmensteigernde Effekte in Höhe von 1 Mrd. bis 2 Mrd. Euro bereits eingerechnet.

Allein die Mehrausgaben bzw. Mindereinnahmen der hier diskutierten Wahlkampfversprechen steigen im Zeitraum 2022 bis 2025 von 37 Mrd. Euro auf 105,1 Mrd. Euro an, was immerhin 20 % des im Finanzplan vorgesehenen Ausgabenvolumens entspricht. Wie bereits beschrieben, hat die Maßnahmenliste dieses Belastungsszenarios keinen Anspruch auf Vollständigkeit, wohl aber für sich genommen ein hohes Maß an politischer Plausibilität. Forderungen nach einem Ausbau des Sozialstaats bleiben dabei ebenso unberücksichtigt wie die noch wesentlich kräftigeren Einkommenssteuerentlastungen, die FDP und CDU/CSU in ihren Wahlprogrammen verankert haben. Und auch die drängenden Infrastrukturfragen sowie die Modernisierung von Wirtschaft und Staat in Zeiten des Klimawandels und der digitalen Transformation, die sich mit Mehrausgaben von etwa 45 Mrd. Euro jährlich beziffern ließen (Bardt et al., 2019), sind in diesen Berechnungen noch gar nicht eingepreist.

Abbildung 2 zeigt im Vergleich mit den im Eckwerteplan des Bundesfinanzministeriums verankerten Projektionen für die Einnahmen und die Ausgaben des Bundes eine klare Abweichung. Inklusive Mütterrente, Übernahme der EEG-Umlage in den Bundeshaushalt, Steigerung der Verteidigungsausgaben, Deckelung der Sozialausgaben bei 40 % sowie Abschaffung des Solidaritätszuschlags bewegt sich das Nettofinanzierungsdefizit weit außerhalb des maximalen Ausgabenspielraums, der schließlich durch die verfassungsmäßig verankerte Schuldenbremse vorgegeben wird. Selbst ein Ausreizen der strukturellen Komponente (0,35 Nettoneuverschuldung des BIP, zwischen 12 Mrd. und 14 Mrd. Euro) würde bei Weitem nicht ausreichen, um der Finanzierungslücke beizukommen. Hinzu kommt, dass die aufgezeigten Ausgabenspielräume (vgl. Abbildung 2) einer mittelfristigen Wachstumsprognose des Bundesfinanzministeriums unterliegen. So wird angenommen, dass das nominale BIP 2021 um

Abbildung 2

**Potenziell steigende Finanzierungslücke im Bundeshaushalt**

in Mrd. Euro Abweichung von der Basislinie in %

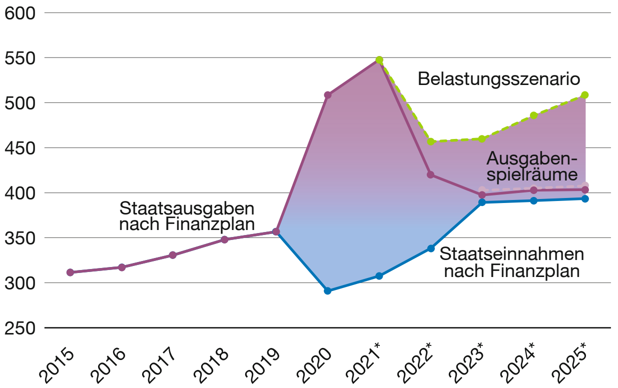

* Werte beziehen sich auf die Kalkulationen aus dem Finanzplan des Bundes von 2020 bis 2024. Belastungsszenario basiert auf eigenen Berechnungen (siehe Tabelle 1)

Quelle: Bundesfinanzministerium (2021b); eigene Berechnungen.

5,6 % und 2022 um 5,5 % wachsen soll, 2023 bis 2025 um weitere 3,1 %[1]. In der aktuellen IW Konjunkturprognose wird von einem realen BIP-Wachstum von 3 % für 2021 und 4 % für 2022 ausgegangen (Bardt et al., 2021a). Diese Schätzung beruht aber auf der Voraussetzung eines erfolgreichen Zurückdrängens der Pandemie. Sollte es zu einem vierten Lockdown in Deutschland im Herbst 2021 kommen, würde dies die deutsche Wirtschaft erneut hart treffen (Grömling und Bardt, 2021).

## Das Steuerloch: absehbare Unwucht

Geht man davon aus, dass die Schuldenbremse auch in der kommenden Legislaturperiode keiner grundsätzlichen Reform unterzogen wird und allenfalls Raum entsteht für einen Investitionsfonds, der grundgesetzkompatibel in eigener Rechtspersönlichkeit konstruiert wird, und geht man ferner davon aus, dass die neue Bundesregierung die oben diskutierten Mehrausgaben tatsächlich umsetzen sowie den Solidaritätszuschlag gänzlich abschaffen möchte, dann steigt unmittelbar der Druck auf die Steuereinnahmen. Auch wenn die strukturelle Komponente der Schuldenbremse voll ausgereizt würde und die Konjunkturkomponente über die Zeit hinweg als neutral betrachtet wird, bliebe von 2022 bis 2025 ein Finanzierungsdefizit von rund 263 Mrd. Euro bestehen. Dieses Defizit beinhaltet jedoch weder die für 2022 vorgesehenen pandemiebedingten und schuldenfinanzierten Mehrausgaben noch die durch einen weiteren pandemiebedingten Wertschöp-

1 Hierbei werden 2 % Inflation ab 2021 mit eingerechnet.

Abbildung 3

**Potenziell steigender Druck auf Steuereinnahmen**

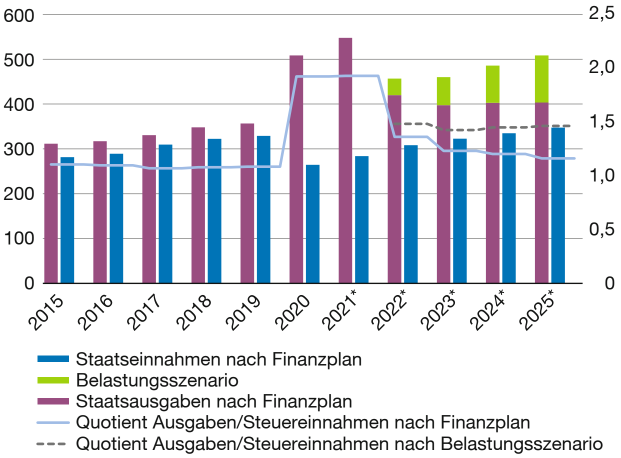

* Werte beziehen sich auf die Kalkulationen aus dem Finanzplan des Bundes von 2020 bis 2024. Werte aus Belastungsszenario basieren auf eigenen Berechnungen (siehe Tabelle 1).

Quelle: Bundesfinanzministerium (2021b); eigene Berechnungen.

fungsverlust potenziell noch entstehenden Steuermindereinnahmen (z. B. bei einem erneuten Lockdown).

Abbildung 3 stellt Steuereinnahmen und Staatsausgaben auf Ebene des Bundes gegenüber. In den Jahren vor der Corona-Pandemie, als der Bundeshaushalt keine Finanzierungsdefizite ausgewiesen hat, lagen die Ausgaben nur leicht über den Steuereinnahmen. Die Quote von Bundesausgaben zu Steuereinnahmen pendelte sich vor 2020 bei rund 1,08 ein. Zusätzliche Staatseinnahmen wie Vermögens- oder Transfereinkommen sowie Einnahmen aus Geldstrafen reichten in dieser Periode aus, um ein Finanzierungsdefizit zu verhindern. Die vielfältigen Hilfen zur Abfederung der wirtschaftlichen Folgen der Corona-Pandemie und der Pandemiepolitik haben die Bundesausgaben aber stark ansteigen lassen; gleichzeitig sind Steuereinnahmen aufgrund des Wirtschaftseinbruchs empfindlich eingebrochen. 2020 und 2021 flossen daher für jeden eingenommenen Steuereuro fast 2 Euro aus der Staatskasse heraus. Diese Ausnahmesituation soll sich nach Finanzplan sukzessive wieder normalisieren. Selbst in dieser konservativen Planung aber würde die Ausgaben-Steuereinnahmen-Quote nicht in die Vor-Corona-Gefilde zurückgeführt werden und auch 2025 noch bei 1,16 liegen. Ein solches Verhältnis zwischen Steuereinnahmen und Staatsausgaben würde eine Nettokreditaufnahme von 10 Mrd. Euro im Jahr implizieren. Preist man die skizzierten, möglichen zusätzlichen Belastungen in den Bundeshaushalt ein, steigt die Quote teils deutlich über 1,4. Damit würde der Staat durchweg über 40 % mehr ausgeben, als er mit Steuereinnahmen einzunehmen vermag.

Auch wenn die vorliegende Belastungsszenarioanalyse einzelne Politikmaßnahmen selektiv herausgepickt und plakativ in den Kontext der künftigen Bundeshaushalte eingeordnet hat, so zeigen die Berechnungen doch eindeutig: Soll die Schuldenbremse unverändert beibehalten werden, wächst der Druck auf die Steuerbelastungen enorm. Schließlich bleibt dem Staat nichts anderes übrig als die Ausgaben-Steuereinnahmen-Quote auf Werte von rund 1,17 zu halten – selbst wenn die Nettoneuverschuldung in der strukturellen Komponente maximal ausgereizt werden soll. Allein 2025 wären dafür rund 100 Mrd. Euro Steuermehreinnahmen notwendig.

## Aus den Schulden herauswachsen?

Die zusätzlichen ausgabenwirksamen Punkte aus den Wahlprogrammen in Kombination mit den avisierten Steuersenkungen stellen für die kommende Legislaturperiode eine große Haushaltsbelastung dar. Insbesondere FDP und CDU/CSU versprechen in ihren Wahlprogrammen deutliche Steuererleichterungen, was mit Blick auf Steuergerechtigkeit und internationale Wettbewerbsfähigkeit gut begründet werden kann; gleichzeitig wollen sie aber grundsätzlich an der Schuldenbremse festhalten. Das von beiden Parteien gern verwendete Zauberwort lautet: „aus den Schulden herauswachsen“ (FAZ, 2021; FDP, 2021b).

Die Staatsschuldenquote bezeichnet das Verhältnis von Staatsschulden zum nominalen BIP und hängt im Wesentlichen von drei Faktoren ab (Busch, 2019): (1) dem Primärsaldo (Budgetsaldo ohne Zinsausgaben), (2) Strom-Bestandsanpassungen (Transaktionen, die den Schuldenstand, nicht aber den Primärsaldo beeinflussen, z. B. Erlöse aus Privatisierungen), und (3) dem Zins-Wachstums-Differential (Differenz zwischen nominalen Zinssatz und der nominalen Wachstumsrate). Wir vernachlässigen Punkt (2) in der folgenden Betrachtung der Entwicklung der Schuldenstandquote und konzentrieren uns auf Punkt (1) und (3).

Abbildung 4 macht – speziell im Hinblick auf das zurückliegende Jahrzehnt – deutlich, dass ein Herauswachsen aus den Corona-Schulden grundsätzlich nicht unplausibel erscheinen muss. Insbesondere, wenn die Neuverschuldung über eine noch längere Periode gestreckt würde, wäre bei der aktuellen Niedrigzinslage ein graduelles Absenken der Schuldenstandquote aufgrund des BIP-Wachstums möglich, allerdings auch voraussetzungsstark, z. B. bei der unterstellten zukünftigen Entwicklung der Beschäftigung am deutschen Arbeitsmarkt (Beznoska et al., 2021; Hüther et al., 2021). Abbildung 4 zeigt zudem, dass auch die aktuelle Bundesregierung davon ausgeht, die Schuldenstandquote bei steigender Nettoneuverschuldung langsam abzusenken. Von ihrem

Abbildung 4

**Erwartetes Absinken der Schuldenstandquote**

in %

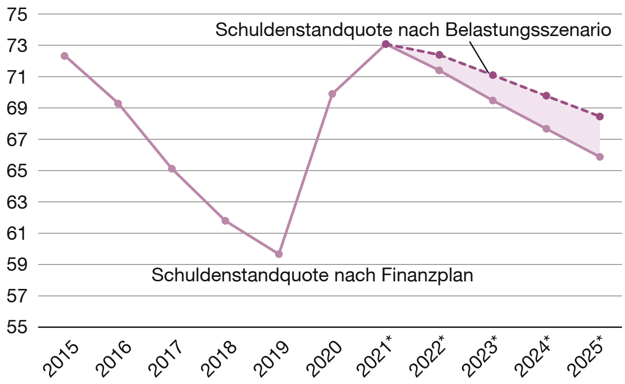

* Werte beziehen sich auf die Kalkulationen aus dem Finanzplan des Bundes von 2020 bis 2024. Belastungsszenario basiert auf eigenen Berechnungen (siehe Tabelle 1).

Quelle: Bundesfinanzministerium (2021b); eigene Berechnungen.

Höchststand von 73 % des BIP im Jahr 2021 soll sie so auf 66 % im Jahr 2025 zurückgeführt werden.

Die Abbildung 4 macht ebenso deutlich, dass auch das Belastungsszenario die qualitative Interpretation kaum verändert. Allerdings führen die eingeplanten Mehrausgaben in den einzelnen Parteiprogrammen zu einer deutlichen Verschlechterung des Primärsaldos (Primärdefizit), was dazu führt, dass die Schuldenstandquote 2025 etwa 2,5 Prozentpunkte höher bei rund 68,5 % liegen würde. Es ist aufgrund der angesprochenen demografischen Ausgangslage mit ihren vielfältigen Auswirkungen auf das Arbeitsangebot und damit auf das Wachstumspotenzial ebenso wenig davon auszugehen, dass die Steuereinnahmen in der nächsten Dekade so stark ansteigen wie nach der Finanzkrise.

Ein Herauswachsen aus den Schulden bleibt in der aktuellen Niedrigzinsperiode auch bei angestiegenen Ausgaben aber grundsätzlich ein plausibler Weg – insbesondere mit Blick auf die derzeitig in Deutschland leicht ansteigenden Inflationsraten (Bardt et al., 2021b). Die gesunkenen Zinsausgaben waren ein wichtiger Faktor, warum die Schuldenstandquote im vergangenen Jahrzehnt in Deutschland so stark gefallen ist. So ist z. B. die Zins-Steuer-Quote, seit 2011 von etwa 11 auf rund 3,3 kontinuierlich gesunken und hat damit den geringsten Wert der vergangenen 50 Jahre erreicht (Beznoska et al., 2021). Die nominalen Zinsausgaben haben sich zwischen 2010 und 2019 von rund 63 Mrd. auf etwa 27 Mrd. Euro mehr als halbiert. Gleichzeitig wird der positive Zinseffekt die Ausgabenseite in den nächsten Jahren nicht so stark entlasten wie im vergangenen Jahrzehnt.

Ein schuldenbremsenkonformes Herauswachsen aus den Schulden in unserem Belastungsszenario ohne weitere Steuereinnahmen würde der Dynamik des BIP allerdings eine ganz andere Dimension abverlangen. Geht man von einer Steuereinnahmeelastizität des BIP von 1,1 aus – wachsen die Steuereinnahmen also etwas stärker als das BIP –, bedürfte es etwa eines utopischen zusätzlichen Wachstums von 16 % des nominalen BIP im Jahr 2023 (gegenüber der 3,1 % der mittelfristigen Projektion im Finanzplan). Diese Zahl ergibt sich aus den 62 Mrd. Euro zusätzlicher Nettobedarfe im Belastungsszenario und der im Finanzplan bereits vorgesehenen 8 Mrd. Euro Nettoneuverschuldung. Ein maximales Ausreizen der Schuldenbremse würde eine um rund 5 Mrd. Euro höhere Nettoneuverschuldung zulassen, sodass sich eine nicht mit Schulden zu deckende Finanzierungslücke von rund 57 Mrd. Euro ergibt. Mit ansteigenden zusätzlichen Ausgaben des Belastungsszenarios würde die Wachstumslücke, die bei einem Einhalten der Schuldenbremse Steuererhöhungen verhindern könnte, bis 2025 sogar auf 26 % ansteigen.

## Wachstumspolitik für Deutschland

Die Kostenkalkulationen eines Belastungsszenarios für die kommende Legislaturperiode machen deutlich, wie unrealistisch eine Umsetzung selbst einzelner im Wahlkampf diskutierter Politikvorschläge bei Beibehaltung der Schuldenbremse und ohne Steuererhöhungen ausfällt. Schon heute ist klar: Die Quadratur des Kreises wird nicht gelingen. Ohne weitere Schuldenfinanzierung oder Steuererhöhungen steht der Bundeshaushalt bereits bei heutiger Planung an seiner verfassungsrechtlichen Kreditaufnahmegrenze. Vorschläge, die Bürger:innen und Unternehmen entlasten, werden sich ebenso wenig umsetzen lassen wie dringende Infrastrukturprojekte zur Modernisierung des Staates sowie zur Bewältigung der Klimakrise und der digitalen Transformation. Wer die Ausgabenwünsche ernsthaft mit Schuldenbremse und ohne Steuererhöhungen umsetzen will, der muss ernsthaft, konsistent und konsequent auf Wachstumsstärkung in der alternden Gesellschaft setzen. Der politische Wille dazu ist nirgends zu sehen. Die notwendigen politischen Schritte werden von den Parteien entweder als Überforderung angesehen oder als Fehlschritt bewertet.

Tatsächlich gibt es mit Blick auf den Bundeshaushalt nur bedingt Einsparpotenziale. Der Sozialhaushalt, der den größten Ausgabenanteil von über 50 % hat, bekommt Druck durch die vielschichtigen Mehrbedarfe einer schrumpfenden und alternden Gesellschaft. Bei den öffentlichen Investitionen bedarf es bei mittel- bis langfristigen Vorhaben mehr nicht weniger Dynamik. Eine Möglichkeit mit Einsparpotenzial könnte sich hingegen bei den Subventionen des Bundes bieten: Vor allem die Klima- und Umweltpolitik wird nach heutiger Planung die

Subventionen des Staates stark ansteigen lassen. Waren es 2019 noch Finanzhilfen und Steuervergünstigungen von rund 25 Mrd. Euro, sollen es 2022 etwas mehr als 47 Mrd. sein (Bundesfinanzministerium, 2021c). Gerade die Klimaschutzmaßnahmen (z. B. Gebäudesanierung) erweisen sich dabei oft mangels Effektivität als sinnvoll. Doch selbst bei signifikanten Kürzungen dieser Subventionszahlungen bliebe der Kern der hier vorgetragenen Argumentation bestehen: Die im Wahlkampf geplanten Belastungen lassen sich unter dem starren Korsett der Schuldenbremse kaum umsetzen.

Zweifellos kann eine wachstumsorientierte Wirtschaftspolitik weitere Spielräume eröffnen, selbst ein deutlich dynamischeres Herauswachsen der deutschen Volkswirtschaft aus dem Corona-Schock wird jedoch kaum die benötigten Steuereinnahmen generieren. Schließlich deutet der Blick in die bundesrepublikanische Steuergeschichte darauf hin, dass das Bundesfinanzministerium große Sprünge nur unter günstigen Arbeitsmarktbedingungen machen konnte. Deutschland steht aber gerade am Ende einer „goldenen Dekade“, deren steigende Arbeitsmarktpartizipation die Konsolidierung des Bundeshaushalts nach der vergangenen Wirtschaftskrise ganz wesentlich ermöglichte. Ein solcher Effekt wird sich – allein demografiebedingt – nicht wiederholen lassen. Bis 2030 schrumpft das Erwerbspersonenpotenzial (Menschen zwischen 20 und 66) in Deutschland voraussichtlich um rund 3,2 Mio. Arbeitskräfte. Eine Ausweitung der im internationalen Vergleich noch immer nicht ausgereizten inländischen Arbeitskräftepotenziale könnte diesen Rückgang zwar ausgleichen (Hüther et al., 2021), findet sich jedoch kaum auf der Wahlkampfagenda der Parteien. Mit Ausgabenversprechen lässt sich der Wahlkampf schließlich eher gewinnen als mit Drängen auf die äußerst unbeliebte Ausweitung von Arbeitszeiten (Brauner et al., 2018; Blömer et al., 2021) – selbst wenn beide Punkte eng verwoben bleiben. Ausnahmen stellen in diesem Kontext potenziell beliebte Politiken dar, die auf den Abbau von unfreiwilliger Teilzeit abzielen oder den Ausbau von Kinderbetreuung adressieren.

Auch mit Blick auf die hohe Bedeutung wachsender Arbeitsvolumen ausländischer Fachkräfte fehlt derzeit die politische Fantasie für eine ernstgemeinte Wachstumsstrategie. Bereits 2018 betrug der in Deutschland erwirtschaftete und auf zugewanderte MINT-Kräfte zurückgehende Wertschöpfungsbeitrag knapp 200 Mrd. Euro. Hinsichtlich der Alterung der Gesellschaft und der damit einhergehenden Fachkräfteengpässe reicht die aktuelle Zuwanderung aber kaum aus, die Arbeitskräftebedarfe der Wirtschaft annähernd zu decken (Anger et al., 2021). Der jährliche Ersatzbedarf an MINT-Kräften bis 2028 liegt derzeit bei rund 291.000 Personen. Selbst abzüglich des neuen erwartbaren Arbeitsangebots aus dem Inland reduziert sich diese Lücke lediglich auf ungefähr 135.000 Personen jährlich (Anger et al., 2021, 28). Geht man davon aus, dass die Zuwanderung in der Post-Pandemieperiode wieder an alter Dynamik gewinnt, ist eine weitere Linderung um jährlich 30.000 bis 36.000 Fachkräfte aus dem Ausland denkbar (Anger et al., 2021, 87 f). Selbst dann bleibt die Lücke beachtlich, zumal eine Rückkehr zur Normalität in der Migrationsfrage derzeit weit entfernt erscheint: Die Migrationshemmnisse, die sich den Unsicherheiten und geschlossenen Grenzen geschuldet während der Corona-Pandemie ergeben haben, bleiben enorm. Momentan leidet gerade die deutsche Wirtschaft unter der seit 2020 stockenden qualifizierten Zuwanderung. Bislang konnte selbst das zurecht gelobte Fachkräfteeinwanderungsgesetz noch keine großen Wirkungen entfalten. Eine weitere Verbesserung der Verfahren beruflicher Anerkennung wäre hierzu unumgänglich (Burstedde et al., 2021). Auch für eine wachstumsorientierte Innovationspolitik ist ein starker Fokus auf qualifizierte Zuwanderung entscheidend. Aktuelle Analysen belegen die wachsende Bedeutung von Zugewanderten für die Innovationstätigkeit der deutschen Wirtschaft. So waren Erfinder:innen mit ausländischen Wurzeln 2018 für mehr als 10 % der deutschen Patenttätigkeit verantwortlich (Kohlisch und Koppel, 2021). Eine weltoffene Zuwanderungspolitik, die entsprechende Fachkräftepotenziale mitdenkt, könnte demnach auch wichtige Impulse für die seit Jahrzehnten darbenden Wachstumsbeiträge des technologischen Fortschritts anstoßen. Sträflich vernachlässigt wurden zudem die lange überfälligen Steuererleichterungen für Forschungs- und Entwicklungsausgaben. Diese würden deutsche Unternehmen im globalen Innovationswettbewerb wieder auf ein Level Playing Field heben.

Zudem erfordert der Strukturwandel zur Dekarbonisierung – zumal nach den verschärften Zielvorgaben für 2035 – und zur digitalen Transformation erhebliche private und öffentliche Investitionen. Das verlangt umfangreiche Infrastrukturinvestitionen des Staates in allen Netzsystemen sowie unternehmerische Investitionen in noch kaum abzuschätzendem Umfang, die stark auf die Vorleistungen des Staates angewiesen sind. Die staatlichen Investitionen sind bisher in den Finanzplanungen nur unzureichend enthalten, vor allem auf Ebene der Länder und Gemeinden fehlt auch eine gemeinsame Perspektive. Das bedroht nicht nur die Bewältigung des Strukturwandels, sondern ebenso die Wachstumsdynamik. Diese ist in der alternden Gesellschaft ohnehin belastet, was weitere Investitionen in das Humankapital und die Beschäftigungsfähigkeit verlangte. All dies legitimiert für sich genommen eine Öffnung der Schuldenbremse für einen Investitionsfonds in mehrjähriger Perspektive („Deutschlandfonds“). Selbst solche innovationsfreundlichen und investitionsstärkenden Politiken wirken jedoch eher auf das mittel- und langfristige Potenzialwachstum der Bundesrepublik und werden die akuten Finanzierungssorgen um den Bundeshaushalt kaum einhegen können. Auch wenn zusätzliche inländische wie ausländische Arbeitskräftepotenziale aktiviert werden und eine innovationsfreundliche Politik technologiegetriebenes Wachstum antreibt, würden die steigenden Steuereinnahmen kaum ausreichen, um die vielen ausgabenintensiven Projekte der kommenden Bundesregierung zu finanzieren. Kurzfristig bleibt als gangbare Lösung das Lockern der Schuldenbremse in Kombination mit einem Abklopfen der Ausgabeposten auf ihre Wachstumspotenziale. Die fiskalpolitische Handlungsfähigkeit jedenfalls bliebe so gewahrt.

## References

[CR1] Anger, C., E. Kohlisch, O. Koppel und A. Plünnecke (2021), MINT-Frühjahrsreport 2021 MINT-Engpässe und Corona-Pandemie: von den konjunkturellen zu den strukturellen Herausforderungen, Gutachten für BDA, BDI, MINT Zukunft schaffen und Gesamtmetall.

[CR2] Bardt, H., S. Dullien, M. Hüther und K. Rietzler (2019), Investitionen ermöglichen! Für eine solide Finanzpolitik, *IW-Policy Paper*, Nr. 10/2019.

[CR3] Bardt H (2021). Verteidigungsausgaben in Deutschland. IW-Trends, 1/2021.

[CR4] Bardt H, Grömling M, Kolev G (2021). Gespaltene Wirtschaft im Wechselbad der Pandemie. IW-Konjunkturprognose Frühjahr 2021. IW-Trends, 1/2021.

[CR5] Bardt, H., M. Diermeier, M. Grömling, M. Hüther und T. Obst (2021b), Lieferengpässe und Preissteigerungen in der deutschen Wirtschaft: Corona oder, here to stay’?, *IW-Report*, 27/2021.

[CR6] BDA-Kommission (2020), Zukunft der Sozialversicherungen: Beitragsbelastungen dauerhaft begrenzen, *Bericht der Kommission*, 29. Juli.

[CR7] Beznoska, M. und T. Hentze (2021), Wahlprogramme zur Einkommensteuer: Alle wollen die Mitte entlasten, *IW-Kurzbericht*, 42/2021.

[CR8] Beznoska, M., T. Hentze und M. Hüther (2021), Zum Umgang mit den Corona-Schulden: Simulationsrechnungen zur Schuldenstandquote, *IW-Policy Paper*, 7/2021.

[CR9] Blömer, M., J. Garnitz, A. Peichl und H. Strandt (2021), Zwischen Wunsch und Wirklichkeit: Unter- und Überbeschäftigung auf dem deutschen Arbeitsmarkt, Bertelsmann Stiftung.

[CR10] Brauner, C., A. Wöhrmann und A. Michel (2018), BAuA-Arbeitszeitbefragung: Arbeitszeitwünsche von Beschäftigten in Deutschland, baua: Bericht.

[CR11] Bundesfinanzministerium (2019), Haushaltsrechnung des Bundes für das Haushaltsjahr 2019, https://www.bundeshaushalt.de/fileadmin/de.bundeshaushalt/content_de/dokumente/2019/ist/HR_2019_Band1.pdf (12. August 2021).

[CR12] Bundesfinanzministerium (2021a), Entwicklung der öffentlichen Finanzen, https://www.bundesfinanzministerium.de/Content/DE/Standardartikel/Themen/Oeffentliche_Finanzen/Entwicklung_Oeffentliche_Finanzen/entwicklung-oeffentliche-finanzen.html (3. August 2021).

[CR13] Bundesfinanzministerium (2021b), Eckwerte des Regierungsentwurfs des Bundeshaushalts 2022 und des Finanzplans 2021 bis 2025, Kabinettsache Datenblatt-Nr.: 19/08180.

[CR14] Bundesfinanzministerium (2021c), Klima- und Umweltziele leiten die zukunftsorientierte Subventionspolitik der Bundesregierung, https://www.bundesfinanzministerium.de/Content/DE/Pressemitteilungen/Finanzpolitik/2021/08/2021-08-18-zukunftsorientierte-sub-ventionspolitik-bundesregierung.html (18. August 2021).

[CR15] Bundesregierung (2020), Finanzplan des Bundes 2020 bis 2024, Unterrichtung durch die Bundesregierung, Drucksache 517/20.

[CR16] Bündnis 90/Die Grünen (2021), Deutschland. Alles ist drin: Bundestagswahlprogramm 2021.

[CR17] Burstedde, A., H. Hickmann, S. Schirner und D. Werner, (2021), Ohne Zuwanderung sinkt das Arbeitskräftepotential schon heute, *IW-Report*, 25/2021.

[CR18] Busch, B. (2019), Die italienische Misere. Ökonomische Strukturprobleme und wirtschaftspolitische Herausforderungen, *IW-Analyse*, Nr. 131.

[CR19] CDU/CSU (2021), Das Programm für Stabilität und Erneuerung: Gemeinsam für ein modernes Deutschland.

[CR20] CSU (2021), Das CSU-Programm: Gut für Bayern. Gut für Deutschland.

[CR21] FAZ (2021), Helge Braun will Schuldenbremse aussetzen, https://www.faz.net/aktuell/politik/inland/corona-kanzleramtschef-braun-will-schuldenbremse-aussetzen-17165322.html?printPagedArticle=true#pageIndex_2 (5. August 2021).

[CR22] FDP (2021a), Nie gab es mehr zu tun: Wahlprogramm der Freien Demokraten.

[CR23] FDP (2021b), Nur die FDP steht für eine Entlastung bei Steuern und Abgaben, https://www.fdp.de/nur-die-fdp-steht-fuer-eine-entlastung-beisteuern-und-abgaben (1. Juli 2021).

[CR24] Feld, L., W. Reuter und M. Yeter, Mustafa (2020), Öffentliche Investitionen: Die Schuldenbremse ist nicht das Problem, *Freiburger Diskussionspapiere zur Ordnungsökonomik*, 20/1.

[CR25] Grömling, M. und H. Bardt (2021), Corona: Vierter Lockdown würde zehn Milliarden Euro kosten, *IW-Nachricht*, https://www.iwkoeln.de/presse/iw-nachrichten/default-146972604b.html (30. August 2021).

[CR26] Handelsblatt (2021), CDU-Kanzlerkandidat Laschet: „Für Steuererleichterung haben wir nicht das Geld“, https://www.handelsblatt.com/politik/deutschland/ard-sommerinterview-cdu-kanzlerkandidat-la-schet-fuer-steuererleichterung-haben-wir-nicht-das-geld/27411664.html?ticket=ST-24159-99WKblpqDbZKfSujwwog-ap2 (2. August 2021).

[CR27] Hentze, T. und G. Kolev (2020), Die Reform des Solidaritätszuschlags vor dem Hintergrund der Corona-Krise, Gutachten im Auftrag der Initiative Neue Soziale Marktwirtschaft.

[CR28] Hüther, M. (2019), 10 Jahre Schuldenbremse — ein Konzept mit Zukunft?, *IW-Policy Paper*, 3/2019.

[CR29] Hüther, M. und J. Südekum (2020), How to re-design German fiscal policy rules after the COVID19 pandemic, Study commissioned by Forum New Economy, *Forum New Economy Working Papers*, Nr. 2.

[CR30] Hüther, M., M. Jung und T. Obst (2021), Arbeitskräftepotenziale der deutschen Wirtschaft: Chancen für Wachstum und Konsolidierung, *IW-Policy Paper*, 10/2021.

[CR31] Kohlisch, E. und O. Koppel (2021), Migration hält Deutschlands stotternden Innovationsmotor am Laufen, *IW-Kurzbericht*, 20/2021.

[CR32] RND (2021), CSU-Pläne zur Mütterrente kosten 4,1 Milliarden Euro jährlich, https://www.rnd.de/politik/csu-plaene-zur-muetterrentekosten-4-1-milliarden-euro-jaehrlich-S5A4NEQT6ZF7ZMPLW7D2F-WOPBA.html (4. August 2021).

[CR33] SPD (2021), Aus Respekt vor deiner Zukunft: Das Zukunftsprogramm der SPD.

[CR34] Tagesschau (2021), Söders doppelte Warnung, https://www.tagesschau.de/inland/btw21/csuprogramm-101.html (2. August 2021).

